# A Study on Dining-Out Habits Among Beijing Residents: A Case of Fast Food

**DOI:** 10.3390/nu17040738

**Published:** 2025-02-19

**Authors:** Zhishan Liu, Wenqiang Chen, Aoran Cui, Kaibiao Gu, Shijun Lu

**Affiliations:** 1 Institute of Food and Nutrition Development, Ministry of Agriculture and Rural Affairs, Beijing 100081, China; 821012450764@caas.cn (Z.L.); cuiaoran2000@163.com (A.C.); 82101235674@caas.cn (K.G.); 2 Section of Integrative Physiology and Metabolism, Joslin Diabetes Center, Department of Medicine, Harvard Medical School, Boston, MA 02215, USA; wenqiang.chen@joslin.harvard.edu; 3 Steno Diabetes Center Copenhagen, 2730 Herlev, Denmark; 4 College of Economics and Management, Shandong Agricultural University, Taian 271001, China

**Keywords:** urban residents, dining out, fast food, nutrient intake

## Abstract

**Background**: With the continuous elevation of living standards, dining-out behavior has become increasingly prevalent among urban residents. The acceleration of lifestyle rhythms has prompted fast food to emerge as a frequently considered dietary option for urban residents when dining out. This study aims to investigate the current status and characteristics of dining-out habits for fast-food consumption among urban residents in Beijing. **Methods**: Urban residents in Beijing were selected using a stratified sampling method to survey restaurants. A database of fast-food items was created, and data were collected through a combination of field observations and qualitative interviews. Nutrient intake from fast food was systematically analyzed. **Results**: Residents consuming fast food while dining out exhibited high per-meal energy intake (737.5 kcal) and protein (44.8 g) consumption; however, the intakes of vitamin A (147.6 μg RAE), vitamin C (22 mg), vitamin E (3.2 mg), and calcium (89.5 mg) were inadequate. Western fast-food meals had higher protein (57.2 g) and sodium (251.5 mg) content compared to Chinese fast food. **Conclusions**: This study provides essential data to guide urban residents toward rational dining choices, offering key insights for the fast-food industry to develop balanced meal options.

## 1. Introduction

With the continuous improvement in living standards and the acceleration of work rhythms in modern life, dining out has become increasingly common among urban residents. The rate of dining out among Chinese urban residents aged 15 and above rose from 26.1% in 2002 to 42.2% in 2012 [1]. Similar trends have been observed in developed countries such as the United States [2], where the proportion of adults dining out increased from 41% in 2000 [3] to 50% in 2010 [4]. In South Korea, a survey conducted in 2015 reported a dining-out rate of 45.1%, highlighting the need for studying dining-out characteristics in urban residents [5].

The choice of dining mode directly influences nutrient intake, dietary structure, and overall health. According to the 2018 China Chronic Disease and Risk Factor Surveillance, more than half of Chinese adults are overweight or obese [6,7]. Studies have shown that individuals who dine out tend to consume more desserts, animal-based meats, and wheat products, while their intake of tubers, vegetables, and fruits is comparatively low. Moreover, meals eaten when dining out typically contain higher levels of energy, fat, and protein—particularly saturated fatty acids, cholesterol, salt, and sugars—as well as lower amounts of dietary fiber, calcium, iron, and vitamin C [8,9]. Diets characterized by high energy, high fat, and low dietary fiber have been widely reported to be closely associated with the development and progression of chronic non-communicable diseases, including obesity, diabetes, hypertension, and hyperlipidemia [10].

Dining out includes a wide range of meal types, including traditional dining, hot pot, buffet, fast food, and so on [11]. Related research has demonstrated that dining out, irrespective of cuisine type, typically results in significantly higher caloric intake compared to home-cooked meals, with fast food consumption exhibiting the most pronounced negative impact on dietary energy balance [12]. Fast food is often associated with a high intake of fats and salts, which can lead to health issues such as hypertension, becoming overweight, and obesity [13]. In addition, fast food often lacks balanced nutritional content [14], which is instead replaced by high levels of protein and fat with insufficient micronutrients. Studies in Australia have shown that fast food results in excessive consumption of fats, saturated fats, sugars, and alcohol compared to home-cooked meals [15,16,17]. While several previous studies in China have addressed dining out among urban residents, few of them have focused on specific meal types [18,19,20]. To fill in this gap, we performed this study to examine the consumption patterns of fast food by urban residents in Beijing, aiming to provide scientific evidence to guide residents in adopting healthy dietary habits.

## 2. Materials and Methods

### 2.1. Research Scope

In this study, fast food refers to meals consumed individually or in small groups, with each person having their own separate meal and a short dining duration. This definition is different from group gatherings or a banquet-style dining pattern.

### 2.2. Participants

Stratified sampling was used to select participants. Beijing’s economic level as a whole decreases from the city center to the periphery, showing certain gradient differences and functional complementarity [21,22]. We divided Beijing into three tiers based on the various residential population and status of economic development: urban areas (Dongcheng, Xicheng, Haidian, Chaoyang, Fengtai, Shijingshan), suburban areas (Mentougou, Fangshan, Tongzhou, Shunyi, Changping, Daxing), and outer suburban areas (Huairou, Pinggu, Miyun, Yanqing). Three districts were randomly selected from each tier. In each district, one Chinese-style and one Western-style fast-food restaurant near commercial areas were selected. Chinese-style restaurants included noodle shops, fast-food outlets serving rice with Chinese dishes, and liangpi (cold noodle) shops. Western-style fast-food restaurants included those serving hamburgers and pizzas. A total of 18 restaurants were surveyed, with 600 adult residents dining out in Beijing randomly selected by professional investigators based on specific fast-food restaurant patronage. Table 1 provides an overview of the sampled participants.

### 2.3. Data Collection Methods

#### 2.3.1. Database Construction

Menus from the surveyed restaurants were analyzed, and the cooked weight (i.e., the edible portion) of fast-food items was measured to the nearest 0.1 g [23,24]. Foods were categorized into a total of 9 groups: staple foods, meats (e.g., pork, beef, poultry), aquatic products, legumes, vegetables (e.g., root, stem, leaf, flower, fruit vegetables, and fungi), fruits, eggs, dairy, and others. Using raw-to-cooked weight conversion factors from the Atlas of Raw and Cooked Food Ratios for Chinese Residents, the raw weights of each ingredient were calculated. Nutrient content was determined based on the China Food Composition Table (6th ed.), resulting in a database of 28,662 entries from 6 fast-food brands [25,26].

#### 2.3.2. Field Investigation

Prior to data collection, professional investigators underwent standardized training, which included repeated practice in weighing dishes and cross-referencing them with restaurant menus. This training enabled them to visually estimate the specific food consumption of participants based on photographs taken on-site, using a fast-food database for comparison. Field observations were conducted by trained investigators who documented food consumption in surveyed diners, including dish types, meal combinations, and food waste. National and provincial technical personnel also provided guidance and conducted inspections to ensure the quality of the survey. One-on-one qualitative interviews were also conducted with diners and restaurant staff to gain insights into consumption behaviors.

### 2.4. Statistical Analysis

Data were recorded in Excel, and nutrient intake was calculated using R 4.4.1 programming. The normality of continuous variables was assessed using the Kolmogorov–Smirnov test in conjunction with Q–Q plots. For variables involved in group comparisons, normality was independently tested within each group. Independent *t*-tests were used to compare two groups for continuous variables, while the analysis of variance (ANOVA) with post-hoc multiple comparisons was applied to assess differences among three or more groups. Statistical significance was set at *p* < 0.05.

## 3. Results

### 3.1. Demographics

This study surveyed 600 adults aged 18 and above, including 324 males (54.0%) and 276 females (46.0%). The demographic distribution is shown in Table 2.

### 3.2. Nutrient Intake During Lunch and Dinner

#### 3.2.1. Lunch

The average energy intake per person during lunch was 744.4 kcal. Male diners consumed 819.5 kcal on average, while females consumed 651.4 kcal. Macronutrient intake for males included 97.2 g of carbohydrates, 47.4 g of protein, and 27.6 g of fat per meal, contributing 46%, 23%, and 31% to total energy, respectively. For females, carbohydrate, protein, and fat intake were 77.3 g, 36.7 g, and 22.4 g, respectively, contributing 47%, 22%, and 31% to total energy. Young adults consumed 768.6 kcal per meal, while middle-aged and older individuals consumed 684.0 kcal. Underweight individuals consumed 92.4 kcal less per meal than those with normal weight, while obese individuals consumed 103.8 kcal more per meal than their normal-weight counterparts. Calcium intake per meal was 85.9 mg for males and 86.9 mg for females, while sodium intake was 179.8 mg and 189.3 mg, respectively. Vitamin C intake per meal was 27.6 mg for young adults and 14.2 mg for middle-aged/elderly individuals, with corresponding vitamin E intakes of 3.2 mg and 2.6 mg. Details for nutrient intake during lunch are presented in Table 3.

#### 3.2.2. Dinner

The average energy intake per person during dinner was 729.7 kcal. Male diners consumed 829.1 kcal on average, while females consumed 619.4 kcal. Macronutrient intake for males included 97.7 g of carbohydrates, 55.6 g of protein, and 24.8 g of fat per meal, contributing 47%, 26%, and 27% to total energy, respectively. For females, carbohydrate, protein, and fat intake were 81.2 g, 37.6 g, and 16.6 g, respectively, contributing 53%, 24%, and 23% to total energy. Young adults consumed 749.3 kcal per meal, while middle-aged and older individuals consumed 666.0 kcal. Individuals underweight consumed 92.4 kcal less per meal compared to those with normal weight, while obese individuals consumed 103.8 kcal more per meal than their normal-weight counterparts. Calcium intake per meal was 90.0 mg for young adults and 102.5 mg for middle-aged/elderly individuals, while sodium intake was 200.7 mg and 205.8 mg, respectively. Vitamin C intake per meal was 22.7 mg for males and 17.1 mg for females, with corresponding vitamin E intakes of 3.8 mg and 2.7 mg. Underweight individuals consumed 1.5 mg less iron per meal compared to those with normal weight. Details for nutrient intake during dinner are presented in Table 4.

### 3.3. Nutrient Intake Differences Between Chinese and Western Fast Foods

#### 3.3.1. Chinese Fast Food

The mean energy intake per meal for Chinese fast food was 719.4 kcal (Table 5). Male diners consumed 788.7 kcal, while females consumed 641.3 kcal. Younger diners consumed 90.4 kcal more energy per meal than older diners. Individuals with normal body weight consumed 27.2 g more carbohydrates per meal than those underweight and 7.8 g less protein than overweight individuals. Vitamin C intake for young adults and older adults was 32.2 mg and 15.2 mg, respectively. Sodium intake was lower for Chinese fast food compared to Western fast food, with 144.9 mg for young adults and 136.5 mg for older adults. Individuals with normal body weight consumed 95.2 μg RAE less vitamin A per meal compared to those who were overweight. Details for nutrient intake per meal for Chinese fast food are presented in Table 6.

#### 3.3.2. Western Fast Food

The mean energy intake per meal for Western fast food was 758.5 kcal (Table 5), with male diners consuming 863.7 kcal and females consuming 628.6 kcal. Younger diners consumed 66.7 kcal more energy per meal compared to older diners. Individuals with normal body weight consumed 131 kcal less energy per meal than overweight individuals and 369.7 kcal less than obese individuals. Vitamin C intake per meal was 18.4 mg for males and 12.8 mg for females, while vitamin E intake was 4.1 mg and 2.7 mg, respectively. Vitamin C intake for younger diners and older adults was 16.1 mg and 15.0 mg, respectively, whereas sodium intake was 249.0 mg and 260.3 mg, respectively. Individuals with normal body weight consumed 2.1 mg and 5.3 mg less iron per meal compared to overweight and obese diners, respectively. Thus, Western fast-food meals exhibited significantly higher protein and sodium content. Details for nutrient intake per meal for Western fast food are presented in Table 7.

## 4. Discussion

In this study, we employed an innovative method of photo recognition to assess the weight, condition, and categories of foods consumed by individuals dining out. This approach enabled the rapid and accurate collection of extensive data on fast-food consumption among a large sample of residents at a relatively low cost. Our findings indicate a positive correlation between energy intake and body mass index (BMI) among participants consuming fast food outside the home. Notably, the protein intake per meal often exceeded the recommended daily allowance, while calcium intake was less than 10% of the daily recommended level. Additionally, Western fast-food meals contained higher amounts of protein and sodium compared to Chinese fast food.

### 4.1. Differences in Nutrient Intake Between Lunch and Dinner

Fast-food consumption among urban residents led to high energy intake, particularly during dinner. Male diners consumed more energy during dinner than lunch, which aligns with studies showing a link between dining-out habits and increased energy consumption. For instance, in Brazil, dining out is associated with becoming overweight and obese [27,28], whereas in Belgium, individuals who dine out have significantly higher energy intake compared to those who do not [3]. Norwegian men frequently dine out for dinner, thus frequently consuming more high-energy foods [29]. Consistently with these studies, Bai et al. found that, among Chinese residents, an increased frequency of choosing fast food when dining out is associated with higher energy intake and an elevated risk of becoming overweight and obese [30].

More excessive energy intake in dine-out meals than home-prepared meals may be attributed to the industrialized cooking methods and the inclusion of high-energy-density foods such as sugary beverages and desserts, as both are commonly included in dining-out habits. Thus, the dining-out environment may predispose individuals to select high-energy-density foods. On the other hand, the higher energy intake observed during dinner could be linked to the daily work routines of urban residents; for instance, many of these individuals have limited time for lunch due to work commitments, leading to a shortened duration for consuming meals, whereas dinner is consumed post-work in a more relaxed setting, potentially increasing food intake [31].

Imbalanced macronutrient intake might occur when consuming fast food. We found that protein intake per meal often exceeds the recommended daily allowance. Previous studies also observed the imbalances in the intake of protein, carbohydrates, and fats among Chinese residents during dining out [32,33,34], in particular with a higher proportion of protein and fat intake [35,36,37], consistent with our findings. This may be due to suboptimal dietary choices when dining out, leading to an insufficient consumption of vegetables and fruits. Moreover, numerous fast-food products emphasize high-protein meat options, potentially encouraging increased meat consumption and resulting in excessive protein intake.

During dine-out fast-food consumption, the intakes of vitamins A, C, and E are notably insufficient, all falling below 25% of the recommended nutrient intake. Among minerals, the intakes of phosphorus, potassium, and zinc are also notably insufficient, while calcium intake is less than 10% of the recommended nutrient intake. Li et al. [38] reported that individuals dining out have daily intakes of vitamins A and C below the recommended levels, consistent with our results. Thus, we recommend that individuals who frequently consume fast food outside of the home should select nutritionally balanced options to ensure an adequate intake of vitamins and minerals, which could prevent hidden hunger [39,40]. Calcium intake among Chinese adults has consistently been at suboptimal levels [41,42]. A study by Huang et al., indicated that in 2015, the average daily calcium intake among Chinese adults was only 345.03 mg, with over 95% of the population consuming less than the Estimated Average Requirement (EAR), making it the most deficient micronutrient [43,44]—a finding consistent with our study. This indicates that inadequate calcium intake is widespread among adults. Research suggests that adequate dietary calcium may help control obesity, diabetes, dyslipidemia, and hypertension [45]. Legumes and dairy products are excellent sources of calcium; consuming dairy can meet nutritional needs and prevent common chronic diseases. It is recommended that adults increase their intake of calcium-rich foods, such as dairy and soy products, when dining out on fast food, and engage in moderate outdoor activities to enhance sunlight exposure [46].

### 4.2. Differences Between Chinese and Western Fast Foods

Western fast-food meals, with excessive consumption of meat, exhibited significantly higher protein levels compared to Chinese-style fast food, and, among obese individuals, energy intake from Western fast food when dining out approached nearly half of their daily energy requirements. Li et al. found that among the three primary macronutrients in fast food, protein and fat levels were disproportionately high, while carbohydrate levels were low [47]. Studies from the United Kingdom also revealed that Western fast food, predominantly meat-based, increased appetite and overeating behaviors, leading to a higher overall daily caloric intake [48,49], American studies have found that the frequency of eating at Western fast-food restaurants is positively correlated with an increase in BMI, and that excessive frequency of dining out at fast food restaurants can lead to becoming overweight and obese [50], consistent with our findings.

The high protein content in Western fast food may be influenced by factors such as menu design, meal combinations, and promotional strategies [51]. Tao et al. noted that Western fast food outperformed Chinese-style fast food in service and marketing, often offering various meal deals and discounts to attract diners. These strategies, coupled with brand image and marketing appeals, particularly attract younger diners who tend to prefer fried meat dishes, resulting in a higher energy intake [52]. It is recommended that Western fast-food providers optimize meal combinations by increasing the availability of vegetables and fruits, such as cherry tomatoes, cucumbers, and lettuce, and consider substituting fried items with grilled options to reduce unnecessary fat intake [53].

We observed that the sodium intake per meal of Western fast food accounts for approximately 20% of the adequate intake, which is higher than Chinese fast food. Relevant research in New Zealand has found that Western fast food leads consumers to consume more sodium [54]. In the United States, from 1997 to 2010, the average sodium content on the menus of Western fast food restaurants increased by 23.4% [55]. Compared with other countries, the sodium intake from Western fast food among urban residents in China is relatively low, which may be related to differences in the popularization of nutritional knowledge, cooking methods, and eating habits in different countries.

In the United States, compared with Chinese fast food, sodium content in main dishes at Western fast-food restaurants is generally high [55]. Reports from Costa Rican fast-food chains also noted high salt levels in chicken products and sauces [56]. Powell et al. highlighted that Western fast food is often characterized by high fat and high salt content, with frequent consumers tending to have lower-quality diets and weaker nutritional awareness [57]. The lack of minerals in Western fast food may be attributed to limited food variety, as meal sets often combine carbohydrates and meats, hindering balanced nutrient intake. In contrast, Chinese-style fast food generally has lower sodium levels, offers a wider variety of ingredients, and employs healthier cooking methods such as steaming, stir-frying, and boiling [58,59].

### 4.3. Study Limitations

The cross-sectional design cannot support causal effects. The sample size is relatively limited, with few overweight and obese individuals, so the findings are representative only of the dietary behaviors of a specific regional group. Our research focuses on fast-food consumption during dining out, whereas in real-life scenarios, dining out encompasses various forms such as traditional meals and hotpot. At the same time, this study only examines the situation of fast-food consumption among urban residents when dining out, without conducting research on rural residents. Future studies should expand the scope to include these diverse dining behaviors.

## 5. Conclusions

This study analyzed fast-food consumption among Beijing residents, highlighting excessive energy intake and unbalanced nutrient profiles. Western fast-food meals exhibited higher protein and sodium content, while Chinese fast food offered more balanced options. These findings provide a guide for urban residents toward healthier dietary choices and support the fast-food industry in optimizing meal compositions.

## Figures and Tables

**Table 1:** Sample overview (number of individuals).

	Male	Female	Total
Urban	168	132	300
Suburban	126	124	250
Outer Suburban	30	20	50

**Table 2:** Demographic characteristics of Beijing residents aged 18 and above [n(r/%)].

Variable	Male	Female
Age Group		
18–29	130 (40.1)	114 (41.3)
30–39	100 (30.9)	103 (37.3)
40–49	64 (19.8)	40 (14.5)
50–60	30 (9.2)	19 (6.9)
BMI Category (kg/m^2^)		
Obese (BMI ≥ 30.0)	9 (2.8)	4 (1.5)
Overweight (25.0 ≤ BMI ≤ 29.9)	35 (10.8)	10 (3.6)
Normal (18.5 ≤ BMI ≤ 24.9)	272 (83.9)	173 (62.7)
Underweight (BMI < 18.5)	8 (2.5)	4 (1.5)
Total	324 (54.0)	276 (46.0)

**Table 3:** Nutrient intake during lunch.

	*n*	Energy (kcal)	CHO (g)	Protein (g)	Fat (g)	Fiber (g)	VitA (μgRAE)	Carotene (μg)	Niacin (mg)	VitC (mg)	VitE (mg)	Ca (mg)	Fe (mg)	Na (mg)
Gender														
Male	173	819.5	97.2	47.4	27.6	2.3	158.5	402.6	12.8	23.0	3.3	85.9	6.2	179.8
Female	140	651.4 *	77.3 *	36.7 *	22.4 *	1.8 *	149.4	479.5	10.2 *	25.0	2.6 *	86.9 *	5.2 *	189.3
Age Group														
18–39	227	768.6	92.7	44.1	25.4	2.2	166.1	463.7	11.9	27.6	3.2	93.3	5.8	189.4
40–60	86	684.0 *	77.3 *	38.8	25.0	1.9	123.5 *	489.5 *	10.8	14.2 *	2.6 *	68.5 *	5.4	170.7 *
BMI (kg/m^2^)														
Obese (BMI ≥ 30.0)	7	857.8	83.7	54.5 ^■^	34.6	2.1	149.3	334.3	14.1	21.4	3.2	90.7	7.9	210.6
Overweight (25.0 ≤ BMI ≤ 29.9)	22	805.7	70.2	52.1 ^●▲^	36.0	2.2	249.8 ^▲●^	465.9	13.9	24.3	3.6	118.5	6.5	242.6
Normal (18.5 ≤ BMI ≤ 24.9)	231	754.0 ^◆^	93.9 ^◆^	41.5 ^▲■^	24.4	2.1	142.3 ^▲^	454.9	11.1	25.1	2.9	85.9	5.6	176.8
Underweight (BMI < 18.5)	53	661.6 ^◆^	72.1 ^◆^	42.1 ^●^	23.4	2.1	165.2 ^●^	375.0	12.6	20.6	2.8	75.2	5.5	188.0

* is a statistically significant difference in nutrient intake between males and females, *p* < 0.05; there is a statistically significant difference in nutrient intake between the 18–39 and 40–60 age groups, *p* < 0.05. BMI multiple comparison analysis: ^◆^ Normal vs. Underweight, *p* < 0.05; ^●^ Overweight vs. Underweight, *p* < 0.05; ^▲^ Overweight vs. Normal, *p* < 0.05; ^■^ Obese vs. Normal, *p* < 0.05.

**Table 4:** Nutrient intake during dinner.

	*n*	Energy (kcal)	CHO (g)	Protein (g)	Fat (g)	Fiber (g)	VitA (μgRAE)	Thiamin (μg)	Niacin (mg)	VitC (mg)	VitE (mg)	Ca (mg)	Fe (mg)	Na (mg)
Gender														
Male	151	829.1	97.7	55.6	24.8	2.6	170.7	0.5	15.7	22.7	3.8	101.2	6.9	236.7
Female	136	619.4 *	81.2 *	37.6 *	16.6 *	2.1 *	105.7 *	0.4 *	10.2 *	17.1 *	2.7 *	83.8 *	5.2 *	163.3 *
Age Group														
18–39	217	749.3	92.3	48.3	21.5	2.4	147.1	0.43	13.5	21.2	3.4	90.0	6.1	200.7
40–60	70	666.0 *	82.0 *	43.3	18.9	2.1	116.4	0.43	11.6	16.2 *	2.8 *	102.5	6.1	205.8
BMI (kg/m^2^)														
Obese (BMI ≥ 30.0)	8	899.6 ^□^	79.4	83.0 ^□■^	28.4	1.9	284.4 ^□■^	0.5	25.8 ^■^	15.0	5.1 ^□■^	112.0	9.3 ^■^	285.9
Overweight (25.0 ≤ BMI ≤ 29.9)	23	827.0 ^●^	96.6	55.5 ^●^	25.0	2.4	185.1 ^●^	0.5	16.4	23.1	3.7 ^●^	88.2	7.1	265.6
Normal (18.5 ≤ BMI ≤ 24.9)	212	737.5 ^◆^	92.7 ^◆^	47.6 ^◆■^	20.3	2.4	137.9 ^■^	0.4	13.1 ^◆■^	19.1	3.3 ^◆■^	96.3	6.2 ^◆■^	197.9
Underweight (BMI < 18.5)	44	618.3 ^◆●□^	73.9 ^◆^	35.4 ^◆●□^	20.7	1.2	106.1 ^●□^	0.4	9.3 ^◆^	19.6	2.4 ^◆●□^	76.9	4.7 ^◆^	176.6

* is a statistically significant difference in nutrient intake between males and females, *p* < 0.05; there is a statistically significant difference in nutrient intake between the 18–39 and 40–60 age groups, *p* < 0.05. BMI multiple comparison analysis: ^◆^ Normal vs. Underweight, *p* < 0.05; ^●^ Overweight vs. Underweight, *p* < 0.05; ^■^ Obese vs. Normal, *p* < 0.05; ^□^ Obese vs. Underweight, *p* < 0.05.

**Table 5:** Comparison of nutrient intake between Chinese fast food and Western fast food.

	*n*	Energy (kcal)	CHO (g)	Protein (g)	Fat (g)	Fiber (g)	VitA (μgRAE)	Thiamin (μg)	Niacin (mg)	VitC (mg)	VitE (mg)	P (mg)	K (mg)	Ca (mg)	Na (mg)
Chinese Fast Food	323	719.4	90.1	34.2	25.5	2.073	132.3	0.4	9.2	27.3	2.9	381.4	602.8	70.8	142.4
Western Fast Food	277	758.5 *	87.8 *	57.2 *	20.5 *	2.356 *	165.4 *	0.4 *	16.0 *	15.9 *	3.5 *	566.9 *	1054.0 *	111.4 *	251.5 *

* is a statistically significant difference in nutrient intake between Chinese fast food and Western fast food, *p* < 0.05.

**Table 6:** Nutrient intake per meal for Chinese fast food.

	*n*	Energy (kcal)	CHO (g)	Protein (g)	Fat (g)	Fiber (g)	VitA (μgRAE)	Thiamin (μg)	Niacin (mg)	VitC (mg)	VitE (mg)	P (mg)	K (mg)	Ca (mg)	Na (mg)
Gender															
Male	171	788.7	97.8	37.1	28.6	2.2	138.7	0.5	9.7	26.9	3.0	410.8	653.2	72.3	151.2
Female	152	641.3 *	81.5*	30.9 *	22.1 *	1.9 *	125.0	0.4 *	8.6	27.8	2.6 *	348.4 *	546.2 *	69.1	132.4
Age Group															
18–39	231	746.0	95.1	34.7	26.0	2.1	144.7	0.44	9.2	32.2	3.0	381.4	599.6	72.3	144.9
40–60	91	655.6 *	78.1 *	32.8	24.3	2.0	100.7 *	0.44	8.9	15.2 *	2.4 *	381.9	613.1	67.0	136.5
BMI (kg/m^2^)															
Obese (BMI ≥ 30.0)	8	718.3	73.6	43.7	28.3	1.7	157.6	0.5	11.9	27.8	2.9	466.7	647.1	61.4	169.1
Overweight (25.0 ≤ BMI ≤ 29.9)	25	755.8	73.0	41.0 ^▲^	34.2	2.0	215.3 ^▲^	0.4	11.0 ^▲^	29.0	3.0	445.7	691.7	83.8	188.6
Normal (18.5 ≤ BMI ≤ 24.9)	235	732.5 ^◆^	97.2 ^◆^	33.2 ^▲^	24.3	2.1	120.1 ^▲^	0.4	8.7 ^▲^	28.6	2.9	372.7	605.3	72.9	134.0
Underweight (BMI < 18.5)	55	646.6 ^◆^	70.0 ^◆^	33.8	26.5	2.0	143.1	0.4	9.9	23.7	2.5	377.0	545.4	57.1	153.1

* is a statistically significant difference in nutrient intake between males and females, *p* < 0.05; there is a statistically significant difference in nutrient intake between the 18–39 and 40–60 age groups, *p* < 0.05. BMI multiple comparison analysis: ^◆^ Normal vs. Underweight, *p* < 0.05; ^▲^ Overweight vs. Normal, *p* < 0.05.

**Table 7:** Nutrient intake per meal for Western fast food.

	*n*	Energy (kcal)	CHO (g)	Protein (g)	Fat (g)	Fiber (g)	VitA (μgRAE)	Thiamin (μg)	Niacin (mg)	VitC (mg)	VitE (mg)	P (mg)	Fe (mg)	Ca (mg)	Na (mg)
Gender															
Male	153	863.7	97.1	67.1	23.7	2.7	192.7	0.5	19.1	18.4	4.1	653.2	6.6	116.3	268.1
Female	124	628.6 *	76.4 *	44.9 *	16.5 *	1.9 *	131.6 *	0.4 *	12.1 *	12.8 *	2.7 *	460.2 *	4.4 *	105.4	231.0
Age Group															
18–39	214	773.3	89.7	58.4	20.7	2.4	169.9	0.4	16.4	16.1	3.6	577.5	5.8	112.4	249.0
40–60	64	706.6 *	81.3 *	52.8	19.5	2.0 *	149.7	0.3 *	14.5	15.0 *	3.1	529.5 *	5.1	108.1	260.3 *
BMI (kg/m^2^)															
Obese (BMI ≥ 30.0)	7	1131.2 ^□■^	94.7	106.0 ^□■^	37.2 ^□■^	2.5	333.3 ^□■^	0.6	31.7 ^□■^	16.8	6.3 ^□■^	977.5	9.8 ^□■^	153.9	364.4
Overweight (25.0 ≤ BMI ≤ 29.9)	20	892.5 ^●^	97.0	70.0 ^●^	25.7 ^●^	2.8	218.6 ^●^	0.5	20.4 ^●^	16.9	4.3 ^●^	681.0	6.6 ^●^	127.0	336.6 ^●^
Normal (18.5 ≤ BMI ≤ 24.9)	208	761.5 ^◆■^	89.0	57.0 ^◆■^	20.3 ^■^	2.4	162.9 ^■^	0.4	15.9 ^■^	15.9	3.5 ^■^	565.8	5.6 ^◆■^	111.1	246.6
Underweight (BMI < 18.5)	42	635.8 ^◆●□^	76.8	45.0 ^◆●□^	16.6 ^●□^	2.1	132.2 ^●□^	0.4	12.7 ^●□^	15.5	2.8 ^●□^	469.3	4.5 ^◆●□^	100.7	221.8 ^●^

* is a statistically significant difference in nutrient intake between males and females, *p* < 0.05; there is a statistically significant difference in nutrient intake between the 18–39 and 40–60 age groups, *p* < 0.05. BMI multiple comparison analysis: ^◆^ Normal vs. Underweight, *p* < 0.05; ^●^ Overweight vs. Underweight, *p* < 0.05; ^■^ Obese vs. Normal, *p* < 0.05; ^□^ Obese vs. Underweight, *p* < 0.05.

## Data Availability

The original contributions presented in this study are included in the article. Further inquiries can be directed to the corresponding author.

## Abbreviations

The following abbreviations are used in this manuscript:

VitA	vitamin A
VitC	vitamin C
VitE	vitamin E
